# NSCs are permissive to oncolytic *Myxoma virus* and provide a delivery method for targeted ovarian cancer therapy

**DOI:** 10.18632/oncotarget.27845

**Published:** 2020-12-22

**Authors:** Yvonne Cornejo, Min Li, Thanh H. Dellinger, Rachael Mooney, Masmudur M. Rahman, Grant McFadden, Karen S. Aboody, Mohamed Hammad

**Affiliations:** ^1^Department of Stem Cell & Developmental Biology, City of Hope, Duarte, CA 91010, USA; ^2^Irell & Manella Graduate School for Biological Sciences, Beckman Research Institute, City of Hope, Duarte, CA 91010, USA; ^3^Department of Information Sciences, Division of Biostatistics at the Beckman Research Institute, City of Hope, Duarte, CA 91010, USA; ^4^Division of Gynecologic Surgery, Department of Surgery, City of Hope, CA 91010, USA; ^5^Biodesign Institute, Arizona State University, Tempe, AZ 85281, USA; ^6^Division of Neurosurgery, City of Hope, Duarte, CA 91010, USA

**Keywords:** oncolytic virotherapy, myxoma, NSCs, ovarian cancer

## Abstract

Despite the development of many anticancer agents over the past 20 years, ovarian cancer remains the most lethal gynecologic malignancy. Due to a lack of effective screening, the majority of patients with ovarian cancer are diagnosed at an advanced stage, and only ~20% of patients are cured. Thus, in addition to improved screening methods, there is an urgent need for novel anticancer agents that are effective against late-stage, metastatic disease. Oncolytic virotherapy is a promising approach; unfortunately, systemic delivery of viruses to tumors remains a major challenge. In this regard, neural stem/progenitor cells (NSCs) with well-established tumor-homing properties may serve as an effective delivery platform for oncolytic viruses. In this study, we tested the efficacy of *myxoma virus* (MYXV), a rabbit-specific poxvirus that has demonstrated efficacy against a variety of tumors, using human and mouse ovarian cancer cell lines. We showed that MYXV effectively lysed ovarian cancer cells *in vitro*, reducing their viability. We also demonstrated that MYXV can infect human NSCs, specifically the clonal HB1.F3.CD21 NSC line. Taken together, these results suggest that NSC-mediated delivery of MYXV may be a promising strategy for achieving more selectively targeted anti-tumor efficacy.

## INTRODUCTION

Chemotherapy and surgery have improved greatly over the last several years, however, ovarian cancer remains lethal with over 21,000 women in the United States expected to be newly diagnosed and almost 14,000 deaths expected this year alone [1–3]. One of the major limitations to effectively treating ovarian cancer is that most patients are diagnosed only after their primary tumor metastasizes to the abdomen and by that time their five-year survival rate is about 45% after Standard Of Care (SOC) treatment [4]. Therefore, it is important to develop new therapies that target metastatic lesions, as well as the primary tumor.

One promising strategy is the use of oncolytic viruses (OVs). These viruses take advantage of faulty mechanisms or pathways commonly found in tumor cells, which allows them to infect and replicate within the tumor cells while sparing healthy cells [5, 6]. The viruses are then able to lyse the tumor cells, irrespective of chemoresistance, and continue infecting neighboring tumor cells to amplify their anti-neoplastic effects. Once the virus reaches normal tissue, no further replication occurs [7]. Additionally, since new tumor antigens are exposed upon lysis, OVs are able to stimulate immune recognition of the cancer cells, further enhancing their therapeutic efficacy [8, 9].

*Myxoma virus* (MYXV), a rabbit-specific poxvirus, has recently been considered for use in oncolytic virotherapy [10–12]. Although it is specific to the European rabbit (causing a lethal disease called myxomatosis) and does not replicate in any non-rabbit normal host cells, it has displayed tumoricidal effect against many human tumor cells [13]. This is thought to occur due to cancer cells: 1) failure to produce appropriate anti-viral responses that can efficiently stop MYXV replication; and 2) constitutive upregulation of pathways associated with cellular transformation that support MYXV replication [14–16].

One major limitation in using OVs to treat cancer is that free viruses can be neutralized by antibodies present in the host patient (due to pre-existing viral immunity), making repeat administration difficult [17]. One way to overcome this limitation is to load the OVs into neural stem/progenitor cells (NSCs). NSCs are tumor-tropic, enabling selective delivery of viruses to tumor cells, minimizing off-target effects. In addition to promoting more effective, localized infection this delivery platform protects the virus from neutralization, thereby increasing viral distribution at tumor sites. We pursued *in vitro* studies in the present study to investigate the pre-clinical efficacy of NSC delivered MYXV on ovarian cancer cells.

## Results

### MYXV showed oncolytic effect for ovarian cancer cells

We incubated human (OVCAR8 and SKOV3) and murine (ID8) ovarian cancer cells with MYXV expressing fluorescence reporter proteins GFP (representative of infection) and TdTr (representative of replication) (vMyx-GFP-TdTomato). These ovarian cancer cells were infected at a multiplicity of infection (MOI) of 10 and time-lapse images captured every 8 h for 48 h post-infection.

**Figure 1:**
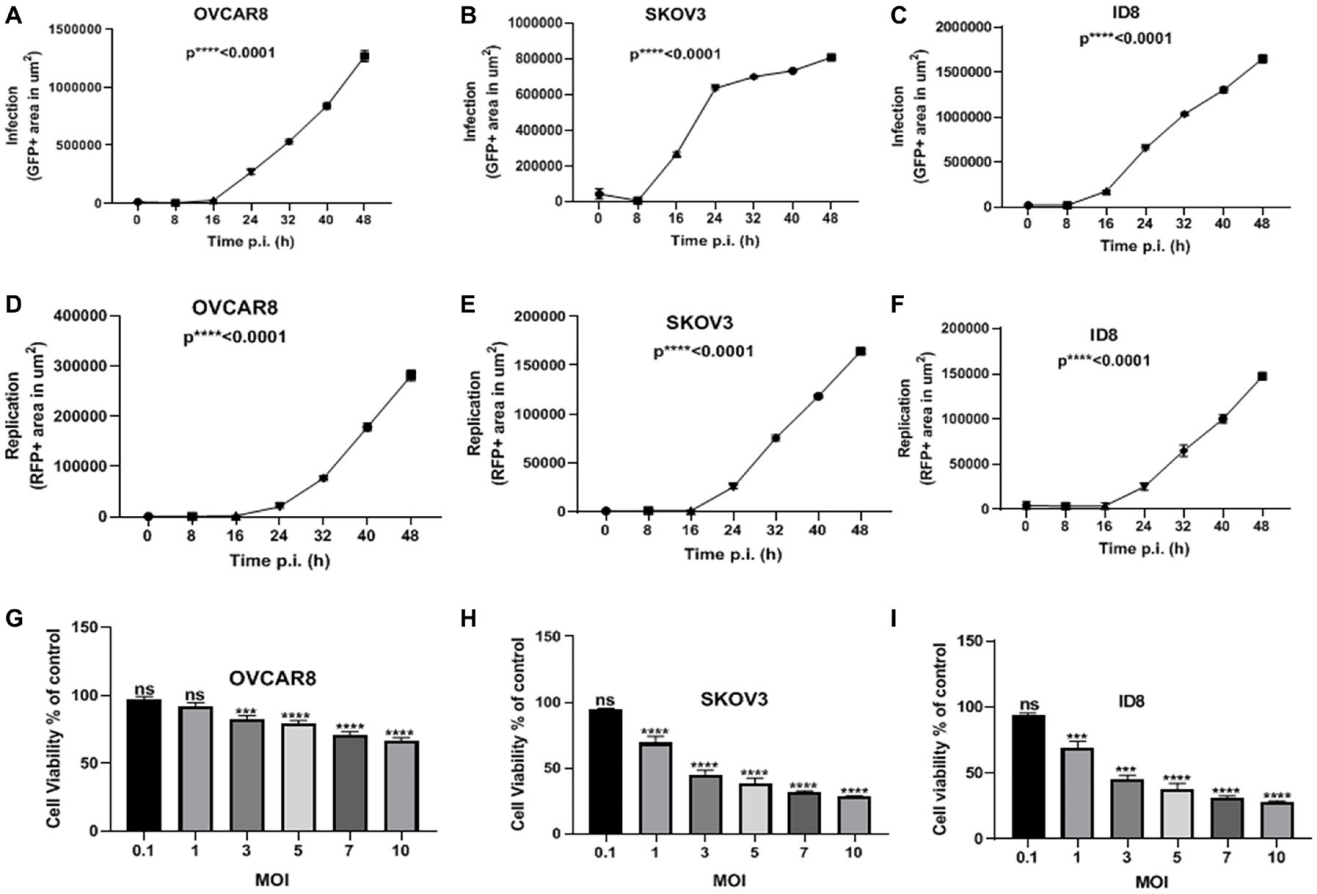
MYXV infects, replicates in, and kills ovarian cancer cells *in vitro*. (**A**) OVCAR8, (**B**) SKOV3, and (**C**) ID8 ovarian cancer cells were treated with vMyx-GFP-TdTr at an MOI of 10, and infection increased significantly over time post-infection. Similarly, the replication of (**D**) OVCAR8, (**E**) SKOV3, and (**F**) ID8 ovarian cancer cells increased significantly over time following infection. (**G**) OVCAR8, (**H**) SKOV3, and (**I**) ID8 were treated with vMyx-GFP-TdTr at multiple MOIs, and 72 h later showed significantly lower viability relative to non-infected control cells. Data are shown as mean ± SEM for at least two repeated experiments. ^***^ *P* < 0.001, ^****^ *P* < 0.0001, as determined by one-way ANOVA comparing infected vs. non-infected cells. ns, no significant difference.

Cells that were not infected were used as the negative control. Total areas (μm^2^/well) of GFP and RFP were calculated and analyzed. All cell lines showed a significant increase in both infection (Figure 1A–1C) and replication (Figure 1D–1F) over time. Next, we infected the same cancer cell lines with vMyx-GFP-TdTr at multiple MOIs and pursued MTS assays to assess the viability of the cells two days after infection. We observed that all cell lines infected with vMyx-GFP-TdTr at MOIs of 3–10 have shown reduced viability compared to non-infected ones (Figure 1G–1I). These data show that vMyx-GFP-TdTr can infect, replicate in, and kill ovarian cancer cell lines.

### NSCs can be infected with vMyx-GFP-TdTr

**Figure 2:**
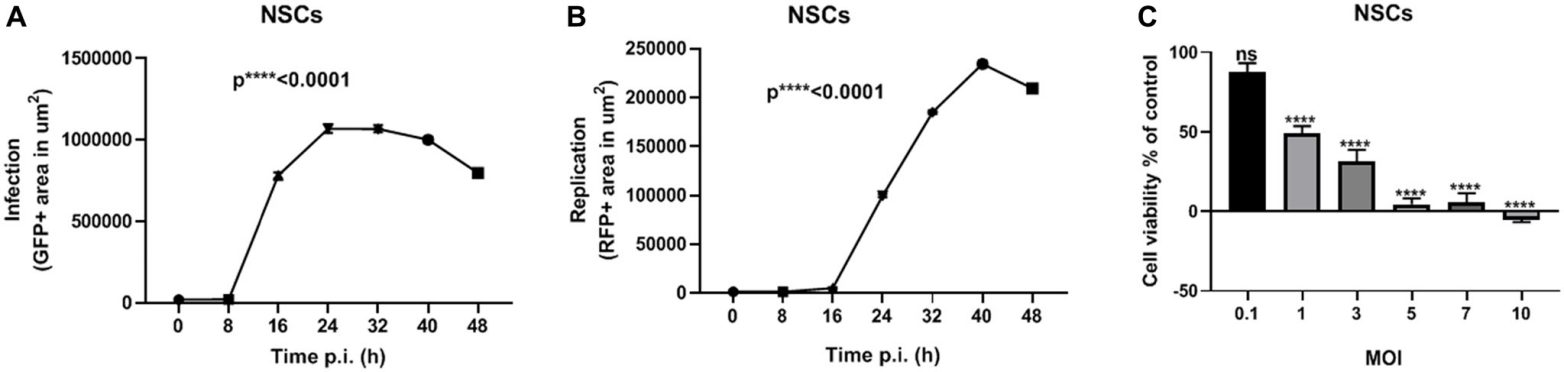
NSCs are permissive to vMyx-GFP-TdTr infection. (**A**) NSCs were treated with vMyx-GFP-TdTr at an MOI of 10, and infection increased significantly over time post-infection. (**B**) Similarly, the replication of vMyx-GFP-TdTr increased significantly over time after infection. (**C**) NSCs were infected with vMyx-GFP-TdTr at various MOIs, and 72 h later showed significantly lower viability relative to non-infected control cells. Data are shown as mean ± SEM for at least two repeated experiments. ^****^ *P* < 0.0001, as determined by one-way ANOVA comparing infected vs. non-infected cells. ns, no significant difference.

In the present study, we used HB1.F3.CD21 NSCs to package MYXV. Specifically, we infected the NSCs with vMyx-GFP-TdTr at an MOI of 10 and captured time- lapse images every 8 h for 48 h post-infection, as we did for the ovarian cancer cell lines. The NSCs showed a significant increase in infection and replication over time (Figure 2A and 2B). We also assessed the effect of vMyx-GFP-TdTr infection on NSCs viability. Seventy-two h after vMyx-GFP-TdTr infection at an MOI of 1, 50% of NSCS were still viable (Figure 2C). This is promising given our previous studies demonstrating that NSCs start seeding virus to tumor sites within 1 hour after i.p. injection in ovarian cancer models [26].

## DISCUSSION

Oncolytic virotherapy has been proven to be safe in many studies [18–21]. However, their efficacy in clinical settings was questionable predominantly because of antiviral immunity responses, inadequate delivery of the virus and poor distribution of the virus to tumor sites [21, 22]. Therefore, it is of utmost importance to utilize a well-established delivery system to enhance the distribution of viruses to tumors and improve their subsequent clinical outcome. Several groups have delivered viruses using autologous cells, including mesenchymal stem cells (MSCs) [19, 23, 24], CD14+-derived monocytes [23] and irradiated tumor cells, interleukin-2 (IL-2)-expanded T cells [25]. Though, these approaches are limited by variability in cell expansion potential, cytogenetic stability, and tumor tropism ability and *ex vivo* viral loading capacity. For example, Mader et al. reported a minimum of two weeks to generate enough amounts of autologous MSCs to treat a patient and around one fifth of the MSC generated showed aberrant karyotypes [24]. MSCs also lose their tumor tropism after 5–6 passages. Hence, we think that using an allogeneic cell line will facilitate clinical translation and long-term scale-up. Furthermore, a cellular delivery vehicle for OVs may considerably advance therapeutic outcomes for treatment regimens that require multiple administrations, especially with the probable following accumulation of neutralizing antibodies against the OV.

With the plausible therapeutic modality of NSC as a delivery platform, we are considering investigating OV cargo with robust anticancer effect. MYXV is a promising OV, as reports have demonstrated that it can infect established human ovarian tumor cell lines [16].

We formerly confirmed that NSCs migrate to ovarian cancer after intraperitoneal (IP) injection into xenograft mouse models with OVCAR8 or SKOV3 peritoneal metastases [17, 26]. Hence, we postulate HB1.F3.CD21 NSC line as a reproducible and predictable carrier vehicle for viral delivery and release kinetics [27]. Indeed, we showed that these NSCs enhance delivery of adenovirus to the peritoneal and brain cancers [17, 27].

Our data indicate that MYXV can kill ovarian cancer cells and that NSCs are permissive to MYXV. These results warrant further investigation of the therapeutic potential of using an NSC platform to deliver MYXV *in vivo*, as well as to study their temporal and spatial distribution, intratumoral retainment, and neutralization mediated by the immune system, particularly after multiple rounds of administration [28]. Future studies should also be conducted to determine the extent to which MYXV provokes a secondary anti-tumor immune response following tumor cell lysis. MYXV can also be manipulated to express therapeutic transgenes, a strategy that performed in several OVs [22, 29].

## MATERIALS AND METHODS

The human ovarian cancer cell line OVCAR8 was kindly gifted by Dr. Carlotta Glackin (City of Hope). We purchased the human ovarian cancer cell line SKOV3 from ATCC. Dr. Katherine Roby (University of Kansas) provided us with the murine ovarian line ID8 then we transduced it to express firefly luciferase. We cultured the ovarian cancer cell lines in RPMI media supplemented with 1% penicillin-streptomycin (Invitrogen), 10% fetal bovine serum (Gemini Bio) and 1% L-glutamine (Invitrogen). Dr. Seung Kim (University of British Columbia, Canada) [30] provided us with the human, v-myc immortalized HB1.F3.CD NSC line. We used this line to generate NSCs that carry MYXV by infecting them with MYXV at multiple MOIs of for 48 h in DMEM (Invitrogen). The media was supplemented with 1% L-glutamine (Invitrogen), 1% penicillin-streptomycin (Invitrogen) and 10% fetal bovine serum (Gemini Bio) and incubated at 37°C in a humidified incubator (Thermo Electron Corporation) containing 6% CO_2_ then harvested. We changed media every 2–3 days and passaged or harvested all cell lines, when they reached confluency at 80% using EDTA solution (Invitrogen) with 0.25% trypsin.

MYXV virus used in the current study has been described in previous studies [31, 32]. In short, MYXV constructs expressing the enhanced GFP and the tandem dimer (td)-Tomato red fluorescent protein (tdTr) were combined in an intergenic location (between the *M135* and *M136* genes) to make vMyx-GFP-tdTr. GFP is under the control of a poxvirus early/late synthetic promoter and tdTr is under the control of a poxviral late (p11) promoter [15].

Ovarian cancer cells (OVCAR8, SKOV3, and ID8) and NSCs were seeded in 96-well plates (5000/well) and treated with vMyx-GFP-TdTr at an MOI of 10. The 96-well plates were placed in an IncuCyte Live-Cell Analysis System (Essen BioScience). Bright field and green and red fluorescence images of the whole field of view for each well were captured every 8 h for 48 h. Images were visualized, and fluorescent areas/well were calculated using IncuCyte software. In another experiment, ovarian cancer cells and NSCs were seeded and allowed to grow overnight at 37°C in an incubator containing 6% CO_2_. The next day, vMyx-GFP-TdTomato was added to each well at various MOIs (0.1, 1, 3, 5, 7 and 10) in 100 μL. MTS assay was conducted as previously discussed [21].

Unless otherwise stated, data are presented as means ± standard error of mean (SEM). One-way ANOVA was used compare virus fluorescence at each time point among the groups with ^*^ *P* < 0.05 considered to be significant.

## Abbreviations

IP: Intraperitoneal
MOI: multiplicity of infection
MSC: mesenchymal stem cell
MYXV: *Myxoma virus*
NSC: neural stem cell
OV: oncolytic virus
PFU: plaque-forming unit
